# Supplementary material for: Level of satisfaction to clinical learning environment and its associated factors among nursing students of public universities in Central Ethiopia
Source: PLoS One. 2025 Jun 27;20(6):e0314240. doi: 10.1371/journal.pone.0314240 (PMC12204464; doi:10.1371/journal.pone.0314240)
Supplement: Attachment — Submitted filename: response to Reviewers.docx [file pone.0314240.s002.docx]

 Please ensure that your manuscript meets PLOS ONE's style requirements, including those for file naming.

- I have read it and I have tried to use as much as possible

We suggest you thoroughly copyedit your manuscript for language usage, spelling, and grammar. If you do not know anyone who can help you do this, you may wish to consider employing a professional scientific editing service.

We note that you have indicated that there are restrictions to data sharing for this study. For studies involving human research participant data or other sensitive data, we encourage authors to share de-identified or anonymized data. However, when data cannot be publicly shared for ethical reasons, we allow authors to make their data sets available upon request.

Before we proceed with your manuscript, please address the following prompts:

a) If there are ethical or legal restrictions on sharing a de-identified data set, please explain them in detail (e.g., data contain potentially identifying or sensitive patient information, data are owned by a third-party organization, etc.) and who has imposed them (e.g., a Research Ethics Committee or Institutional Review Board, etc.). Please also provide contact information for a data access committee, ethics committee, or other institutional body to which data requests may be sent.

b) If there are no restrictions, please upload the minimal anonymized data set necessary to replicate your study findings to a stable, public repository and provide us with the relevant URLs, DOIs, or accession numbers. Please see  for guidelines on how to de-identify and prepare clinical data for publication. For a list of recommended repositories, please see. You also have the option of uploading the data as Supporting Information files, but we would recommend depositing data directly to a data repository if possible.

Please update your Data Availability statement in the submission form accordingly.

**Response:-**all the data used for this research were included in the document. And the the raw data can be accessed from corresponding author at a reasonable request.

The manuscript has 4 tables, and for all of them I suggest:
1- synthesize the information to make it more appealing and for clearer reading;
2- reduce information from the table titles (if absolutely necessary, place it as a table note -footnote);
3- Do not use redundant information in the table titles or the content of the table vs. text

**Response: -** Revised accordingly

Reviewers' comments:
Reviewer's Responses to Questions

**Comments to the Author**

1. Is the manuscript technically sound, and do the data support the conclusions?

The manuscript must describe a technically sound piece of scientific research with data that supports the conclusions. Experiments must have been conducted rigorously, with appropriate controls, replication, and sample sizes. The conclusions must be drawn appropriately based on the data presented.

Reviewer #1: Yes

2. Has the statistical analysis been performed appropriately and rigorously?

Reviewer #1: Yes

3. Have the authors made all data underlying the findings in their manuscript fully available?

The [PLOS Data policy](http://www.plosone.org/static/policies.action#sharing) requires authors to make all data underlying the findings described in their manuscript fully available without restriction, with rare exception (please refer to the Data Availability Statement in the manuscript PDF file). The data should be provided as part of the manuscript or its supporting information, or deposited to a public repository. For example, in addition to summary statistics, the data points behind means, medians and variance measures should be available. If there are restrictions on publicly sharing data—e.g. participant privacy or use of data from a third party—those must be specified.

Reviewer #1: Yes

4. Is the manuscript presented in an intelligible fashion and written in standard English?

PLOS ONE does not copyedit accepted manuscripts, so the language in submitted articles must be clear, correct, and unambiguous. Any typographical or grammatical errors should be corrected at revision, so please note any specific errors here.

Reviewer #1: Yes

5. Review Comments to the Author

Please use the space provided to explain your answers to the questions above. You may also include additional comments for the author, including concerns about dual publication, research ethics, or publication ethics. (Please upload your review as an attachment if it exceeds 20,000 characters)

Reviewer #1: Level of Satisfaction to Clinical Learning Environment and Its Associated Factors among Nursing Students of Public Universities in Central Ethiopia

Congratulations on choosing this topic; it is pertinent and relevant for understanding the clinical learning of students and the conditions that influence their satisfaction. Utilizing a cross-sectional study is highly appropriate for deepening knowledge in this area.

Abstract
Regarding the abstract, the objective is concise and appropriate to the topic and nature of the study. It summarizes the most relevant aspects without exceeding 300 words. It clarifies who the study participants are and the context.

Introduction
The introduction is well-supported by reference authors. The references are extensive, current, and the topic is explored adequately.

Materials and Methods
1. Clarifies the type of study;
2. Identifies the type of context;
3. Clearly explains the criteria for sample identification and inclusion;
4. Ethical considerations for a study of this nature are clear.

**Response:-. Type of the study**: - An institutional based cross-sectional study involving was conducted

**Response: -** Clearly explains the criteria for sample identification and inclusion;-

**Response: - Study population**

All randomly selected second year and third year undergraduate nursing students who are studying at universities found in Central Ethiopia.

**Response:-Sampling unit**

Individual Nursing student who was selected during the data collection period.

**Response:-Inclusion criteria**

All undergraduate nursing students of second year and third year as well as those participated at least once in clinical practice.

**Response:-Exclusion criteria**

Fourth year nursing student due to educational leave at the time of the study.

**Limitations and Constraints**

It is essential to present the study's limitations and constraints, as well as the benefits of these findings for the education and preparation of nurses.

**Response: -** This study only looks at the quantitative data; therefore, additional insights into the qualitative responses from the students regarding their opinions and/or perceptions of the CLE could be gained by using a mixed methodology akin to other pertinent studies. The impact of the CLE on students' learning and development cannot be solely determined by looking at student satisfaction. Other intriguing and unknown variables may surface in such a mixed methodology. The study was limited to only public universities, the findings from a private facility may be different. Furthermore, the questionnaire contained Likert scale questions that offered a choice to participants to be indecisive when responding. This aspect might have affected the accuracy and validity of the study findings.

**Response: -** **Benefits of these findings for the education and preparation of nurses.**

The finding of this study will be help nursing department, minister of education and nurse association to understand the nursing student’s satisfaction with clinical learning environment and factors that affect nurse students’ satisfaction and to design strategy to enhance factors positively influence on nurses ‘student satisfaction as well as improve factors that contribute for dissatisfaction with clinical learning environment. In addition, the knowledge generated from this study will be enriched literatures available on the issue and aid for further a researcher who has an interest in conducting researches on this topic.

6. PLOS authors have the option to publish the peer review history of their article ([what does this mean?](https://journals.plos.org/plosone/s/editorial-and-peer-review-process#loc-peer-review-history)). If published, this will include your full peer review and any attached files.
If you choose “no”, your identity will remain anonymous but your review may still be made public.

**Do you want your identity to be public for this peer review?** For information about this choice, including consent withdrawal, please see our [Privacy Policy](https://www.plos.org/privacy-policy).

Reviewer #1: No

While revising your submission, please upload your figure files to the Preflight Analysis and Conversion Engine (PACE) digital diagnostic tool, <https://pacev2.apexcovantage.com/>. PACE helps ensure that figures meet PLOS requirements. To use PACE, you must first register as a user. Registration is free. Then, login and navigate to the UPLOAD tab, where you will find detailed instructions on how to use the tool. If you encounter any issues or have any questions when using PACE, please email PLOS at [figures@plos.org](mailto:figures@plos.org). Please note that Supporting Information files do not need this step.

\# I have done it accordingly
