# Supplementary material for: Level of satisfaction to clinical learning environment and its associated factors among nursing students of public universities in Central Ethiopia
Source: PLoS One. 2025 Jun 27;20(6):e0314240. doi: 10.1371/journal.pone.0314240 (PMC12204464; doi:10.1371/journal.pone.0314240)
Supplement: Attachment — Submitted filename: Response_to_Reviewers_auresp_2.docx [file pone.0314240.s003.docx]

**October 24, 2024**

**Editorial Department**

**PLOS ONE**

**Re: Manuscript Number: PONE-D-24-08053R1**

**Dear Editorial staffs;**

Thank you for the opportunity to submit our manuscript “Level of Satisfaction to Clinical Learning Environment and Its Associated Factors among Nursing Students of Public Universities in Central Ethiopia" for consideration for publication in PLOS ONE.

We have carefully addressed the reviewers’ comments in our responses and incorporated the related changes into the manuscript. These responses are detailed in the enclosed Response to Reviewers; for convenience, reviewers’ comments are provided in black font and our responses are in green. Furthermore, changes made in the manuscript are highlighted by yellow color. We grateful to the reviewers for their valuable comments. We believe that the revised manuscript is much stronger as a result of their feedback.

Finally, we appreciate the level of insight and detail provided by these constructive comments.

Thank you again for your consideration of this manuscript.

Sincerely!

**Samuel Abate Walda**

**Editor Comments:**

Dear authors

Please respond to all suggestions made by reviewers, in order to overcome the weaknesses highlighted in the manuscript, especially those highlighted by reviewer 2.

Response: We appreciate the valuable suggestions and recommendations given to us by the editor and the reviewers. In this revision, due attention was given to every section, especially the comments given by reviewer 2. All comments point by point were addressed and highlighted by yellow color in the revised content in the text. Thank you!

**Reviewer #2:**

1. The starting question of the study is not clear when reading the document, nor is the objective. I suggest that these items, which are essential to all studies, be revised.

Response: Thank you for your suggestions. This has now been corrected as suggested. Under the introduction section, page 5, paragraph 3, lines 126-139.

2. The presentation of the results is descriptive, which makes it confusing. My suggestion is that it should be presented using tables and graphs that are appealing for reading and interpreting the data, thus making it easier to understand.

Response: Thank you for your suggestion and comment. This has now been corrected as suggested. Under references section page 13, line 343 and page 11 line 303, page 12, line 321, page 12 line 325.

3. The table titles are too big, they need to be improved

Response: We sincerely acknowledged and improved it. This has now been corrected as suggested. Under result section, Page 21-26, line 527-539.
